# Morpho‐Physiological Traits and Dehydration Tolerance of High‐Altitude Andean Wetland Vegetation in the Chilean Atacama Region

**DOI:** 10.1002/pei3.70038

**Published:** 2025-04-01

**Authors:** Dariel López, Patricia L. Sáez, Lohengrin A. Cavieres, Fernanda C. Beveridge, Felipe Saavedra‐Mella, León A. Bravo

**Affiliations:** ^1^ Laboratorio de Fisiología y Biología Molecular Vegetal, Departamento de Ciencias Agronómicas y Recursos Naturales. Facultad de Ciencias Agropecuarias y Medioambiente Universidad de La Frontera Temuco Chile; ^2^ Instituto de Ecología y Biodiversidad (IEB) Concepción Chile; ^3^ Departamento de Botánica, Facultad de Ciencias Naturales y Oceanográficas Universidad de Concepción Concepción Chile; ^4^ Sustainable Minerals Institute, International Centre of Excellence The University of Queensland Santiago Chile

**Keywords:** alpine, conservation, drought, ecological strategies, plant functional traits, salinity

## Abstract

High‐altitude wetlands of the Andes (HAWA) are unique ecosystems influenced by substrate conditions and reliant on consistent water supply from precipitation, runoff, groundwater, and glacial melting. Considering the diverse ecosystem services provided by HAWAs and the increasing threat these ecosystems face from natural and anthropogenic factors, such as drought, land‐use change, and climate change, it is crucial to conduct a comprehensive assessment of their vulnerability. In this study, we characterized the functional trait spectrum of dominant plant species within the Salar de Pedernales, Quebrada Leoncito (Leoncito) and Río Negro HAWAs and explored the relationships between these traits and key environmental variables. Our results revealed significant variation in plant species based on traits such as leaf dry matter content (LDMC), specific leaf area (SLA), relative water content (%RWC), and leaf thickness. Species were primarily differentiated by LDMC and SLA. Plants from Salar de Pedernales had higher δ13C values, indicating higher water‐use efficiency (WUE) compared to those in tributaries like Leoncito and Río Negro. A positive correlation between stomatal conductance and CO_2_ assimilation was found, with the Salar de Pedernales plants showing high WUE despite these plants exhibiting similar photosynthetic rates. Foliar nitrogen percentage and δ^15^N values indicated nitrogen availability could be influenced by microbial activity and salinity levels. Higher salinity in the Salar de Pedernales may inhibit microbial activity, resulting in higher δ^15^N values. At the community level, decreased SLA correlated with higher δ^13^C values, suggesting less carbon discrimination and higher WUE in the Salar de Pedernales plants. While HAWA plant species have adapted to their environment, their limited dehydration tolerance makes them vulnerable to future hydrological changes. Understanding these responses forms a basis to develop effective conservation and management strategies for HAWAs.

## 1 Introduction

High‐altitude wetlands of the Andes (HAWA) constitute unique ecosystems characterized by their “azonal” condition, primarily influenced by substrate conditions rather than climatic zones (Ruthsatz 1993, 2012; Squeo et al. 1993). These environments rely on a consistent water supply derived from various sources, including precipitation, surface runoff, groundwater, and glacial or snow melting (Squeo et al. 2006). However, the hydrological regime is not the sole determinant of plant community composition, as the influence of dissolved salts and heavy metals is also significant (Ginocchio et al. 2008). HAWA provide a suite of invaluable ecosystem services, including hydrological regulation, water purification, habitat provision, and carbon sequestration (Ahumada et al. 2011). Nevertheless, these ecosystems are increasingly threatened by a combination of natural and anthropogenic factors, including drought, land‐use change, and climate change. In recent decades, the global transition to renewable energy has markedly increased the demand for critical minerals, many of which are concentrated in the high Andean Altiplano, where HAWA are prevalent. This demand, driven by the necessity for key materials such as lithium, copper, and other strategic elements integral to battery technologies and wind energy systems, has intensified environmental pressures in these regions (Romero et al. 2017; Prieto et al. 2019). The compounding effects of climate change further exacerbate these challenges, as alterations in the precipitation patterns and temperature regimes induce variability in hydrological cycles. These climatic shifts have amplified water scarcity and disrupted the hydrological balance of HAWA, potentially heightening the susceptibility of these ecosystems to ecological stress.

To comprehensively assess the vulnerability of HAWA to these converging stress factors, a functional trait‐based approach is essential. Plant functional traits, such as those from the leaf or plant economy spectrum, are the phenotypic expressions of species' adaptations to their environment, providing critical insights into plant responses to environmental changes (Díaz and Cabido 2001; McGill et al. 2006). By examining the distribution of functional traits within plant communities, we can gain a deeper understanding of ecosystem processes, responses, and resilience to disturbance (Adler et al. 2013; Garnier et al. 2015; Herben et al. 2018; Niklas et al. 2023). The leaf economic spectrum traits include two easily assessed traits: specific leaf area (SLA) or its inverse leaf mass per area (LMA), and leaf dry matter content (LDMC). Both traits are critical to our understanding of terrestrial ecosystem dynamics, nutrient cycles, responses to current global climate change, and the evolutionary trajectories of foliage form and function (Niklas et al. 2023). Additionally, variations in the stable isotopic composition of C (δ^13^C) and N (δ^15^N) in plants and soils are the result of fractionation processes occurring during the ecosystem exchange of carbon and nitrogen. Thus, δ^13^C and δ^15^N can serve as valuable indicators of ecosystem state and provide useful insights on how these systems respond to biotic and abiotic factors (Gerschlauer et al. 2019).

This study aimed to characterize the functional trait spectrum of dominant plant species within the Salar de Pedernales, Leoncito, and Río Negro HAWAs and to explore the relationships between these traits and key environmental variables. The findings will contribute to the development of predictive models and inform the implementation of effective conservation and management strategies for these vulnerable ecosystems.

## 2 Materials and Methods

### 2.1 Study Sites and Selected Species

The study area corresponds to the endorheic basin of Salar de Pedernales, located about 4000 m.a.s.l. in the Atacama region, with an extension of 3596 km^2^. Three main rivers flow into this basin: Río Juncalito with its main tributary Río Negro, Río La Ola, and Río Leoncito. A large part of the waters of Río Juncalito flow artificially towards Río Leoncito, which contributes to Río La Ola, whose flow is dammed in the La Ola dam before reaching the Salar de Pedernales. The electrical conductivity of the waters varies from a minimum of around 40.7 μS/cm at the head of the rivers, gradually increasing as it descends towards the lower part of the basin, finding the maximum in the Salar de Pedernales of 273 mS/cm, where surface waters tend to be dominated by sodium chloride, although local calcium sulfate waters may occur (Pérez et al. 2019).

**FIGURE 1:**
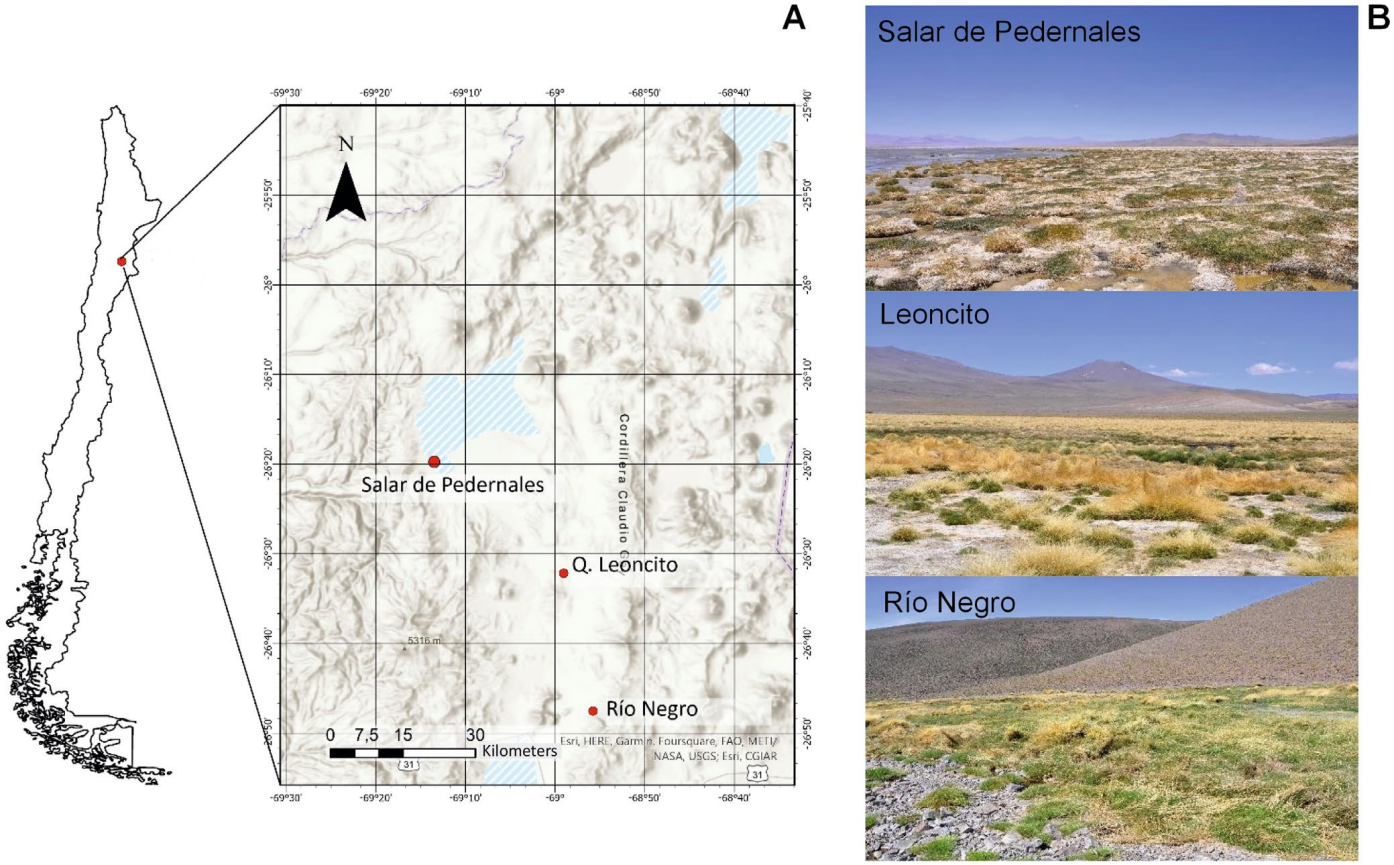
Location of the study site in the Atacama region (A), and pictures of the studied habitats Salar de Pedernales, Leoncito and Río Negro HAWAs (B).

The study was carried out at three locations (Figure 1): the western region of the Salar de Pedernales HAWA (UTM: 7089642.79; 476477.27) with an area of 37.48 ha. This site had the lowest floristic diversity. The vegetation is predominantly composed of *Puccinellia frigida* (Phil.) I.M. Johnst., *Triglochin concinna* Burtt Davy, and *Zameioscirpus atacamensis* (Phil.) Dhooge & Goetgh., growing on the margins of small lagoons surrounded by a crust of gypsum and halite. The second study location corresponds to the Leoncito HAWA (UTM: 501578.251; 7064950.159) with an area of 46.22 ha. In this site, fresh water emerges and flows into a stream that moves eastwards. This HAWA has the greatest floristic diversity, dominated by species of *Deyeuxia* Clarion ex P.Beauv., *Scirpus* L., and *Hordeum* L. genus, and species such as *P. frigida* *and Oxychloe andina* Phil., among others. Finally, the Río Negro HAWA (UTM: 506997.961; 7036610.149) with a surface area of 2.97 ha is located in the bed of a ravine surrounded by low hills. It has an intermediate floristic diversity, with vegetation dominated by species of *Deyeuxia* genus and *O. andina* , among other less abundant species. A total of eight dominant native species from four different families were selected for this study across the three locations sampled (Table 1).

**TABLE 1:** Nine Chilean native species selected for this study, which are dominant in the three selected study sites: Salar de Pedernales, Leoncito and Río Negro HAWAs.

Species	Family	Study site
*Deyeuxia eminens*	Poaceae	Leoncito, Río Negro
*Deyeuxia velutina*	Poaceae	Leoncito
*Deyeuxia* sp.	Poaceae	Río Negro
*Hordeum* sp.	Poaceae	Leoncito
*Oxychloe andina*	Juncaceae	Leoncito, Río Negro
*Puccinellia frigida*	Poaceae	Leoncito, Salar de Pedernales
*Scirpus* sp.	Cyperaceae	Leoncito
*Triglochin concinna*	Juncaginaceae	Salar de Pedernales
*Zameioscirpus atacamensis*	Cyperaceae	Salar de Pedernales

Meteorological data since 2011 was obtained from Río la Ola in Vertedero (station number 03022001–3) (DGA 2021), located 28 km south of Salar de Pedernales, 8 km north‐west of Leoncito HAWA, and 36 km north of Río Negro HAWA. Meteorological data show that the maximum temperature occurs between January and April (between 20°C and 31°C), whilst the minimum occurs between May and August (between −12°C and −18°C), with an average daily variation greater than 25°C. The precipitations (liquid + solid) have an annual average of 140 mm (Pérez et al. 2019).

### 2.2 Morpho‐Physiological Traits

Different morphophysiological traits were measured for 6 plants of each species: *plant size*, estimated as the projection of the basal area of the canopy, assuming an elliptical shape for each plant; *plant height*, determined as the distance from the ground to the tallest photosynthetic tissues; *leaf thickness*: estimated with a digital micrometer; *leaf chlorophyll content*: estimated with a portable chlorophyll content meter (CCM300‐Opti‐Science) on 3–4 leaves for each individual; *leaf mass per unit area* (LMA): estimated as the ratio between the dry biomass of a leaf and the area of the same fresh leaf and their opposite specific leaf area (SLA); and *leaf dry matter content* (LDMC), determined as the dry leaf weight divided by the saturated fresh leaf weight. For both LMA and LDMC, 2–10 well‐developed fresh leaves were weighed per individual using a microbalance (uncertainty ±1 μg). The leaf area was estimated with a digital scanner and the ImageJ software. Then, the leaves were transported in silica gel to the laboratory where they were dried in an oven at 60°C for 72 h to determine the dry weight.

### 2.3 Stable Isotope Analyses

Stable isotopes of ^15^N and ^13^C from 5 to 6 plants per species were analyzed following the procedure described by Díaz et al. (2016). The leaf samples were carefully collected in the field using gloves and immediately introduced into paper bags for subsequent drying at 70°C for 72 h. The samples were sent to the Laboratory of Biogeochemistry and Applied Stable Isotopes (LABASI) of the Department of Ecology, Pontificia Universidad Católica, Chile, where stable isotopes were determined using a Thermo Delta V Advantage Isotope Ratio Mass Spectrometer (IRMS) coupled to a Flash2000 elemental analyzer.

### 2.4 Dehydration Tolerance Test

The effect of leaf dehydration on photochemical activity was studied using the method described by López‐Pozo et al. (2019) with modifications. In summary, the physiologically active leaves of 3 individuals per species were exposed to dehydration conditions inside Falcon tubes (Falcon Test) balanced at three different relative humidities (80%, 50% and < 10%), and subsequently rehydrated. The maximal photochemical efficiency of PSII (*Fv/fm*) was measured using a Mini‐PAM fluorimeter (Walz, Effeltrich, Germany) in leaves before dehydration and after rehydration, and the level of damage suffered by the dehydration was determined as the photoinactivation percentage (PI%). The results are presented as the relative water content (RWC) percentage at which 50% photoinactivation of the leaf tissue occurs (RWC_50_).

### 2.5 Light‐Saturated Net Photosynthesis

Leaf gas exchange measurements were performed in situ with a portable Li‐6400XT infrared gas analyzer (IRGA) (LI‐COR Inc. Lincoln, NE, USA). Six individuals of each species were used to measure foliar net photosynthesis (A_N_), stomatal conductance (g_s_), and transpiration rate (E). The measurements were made at 1500 μmol photons m^−2^ s^−1^, at a block temperature of 20°C, with a relative humidity between 50% and 60%, and with a flow of 300 mL s^−1^. We tried to cover the entire IRGA plant leaf cuvette when possible. When this did not happen due to the leaf size, a correction was made by the ratio of cuvette area/leaf area. The instantaneous water use efficiency (WUEi) was obtained by dividing A_N_ by E for each individual.

### 2.6 Statistical Analysis

A principal component analysis (PCA) was performed with all the traits measured to assess the trait differentiation among species. For this, data were standardized, and the PCA extraction was done with a covariance matrix. PCA analysis was done on the platform MetaboAnalyst 6.0 (Pang et al. 2024).

The differences among species on RWC_50_, A_N_, and WUE_i_ were assessed using one‐way ANOVA tests, and the Duncan test was used to determine those differences. The Pearson correlation test assessed the significant linear relations between the δ^15^N and foliar nitrogen percentage, the δ^13^C and LMA, stomatal conductance and A_N_, and RWC_50_ and A_N_. The analyses were performed with the software Statistica 8.0 (Stat Soft Inc. Tulsa, OK, USA).

## 3 Results

**FIGURE 2:**
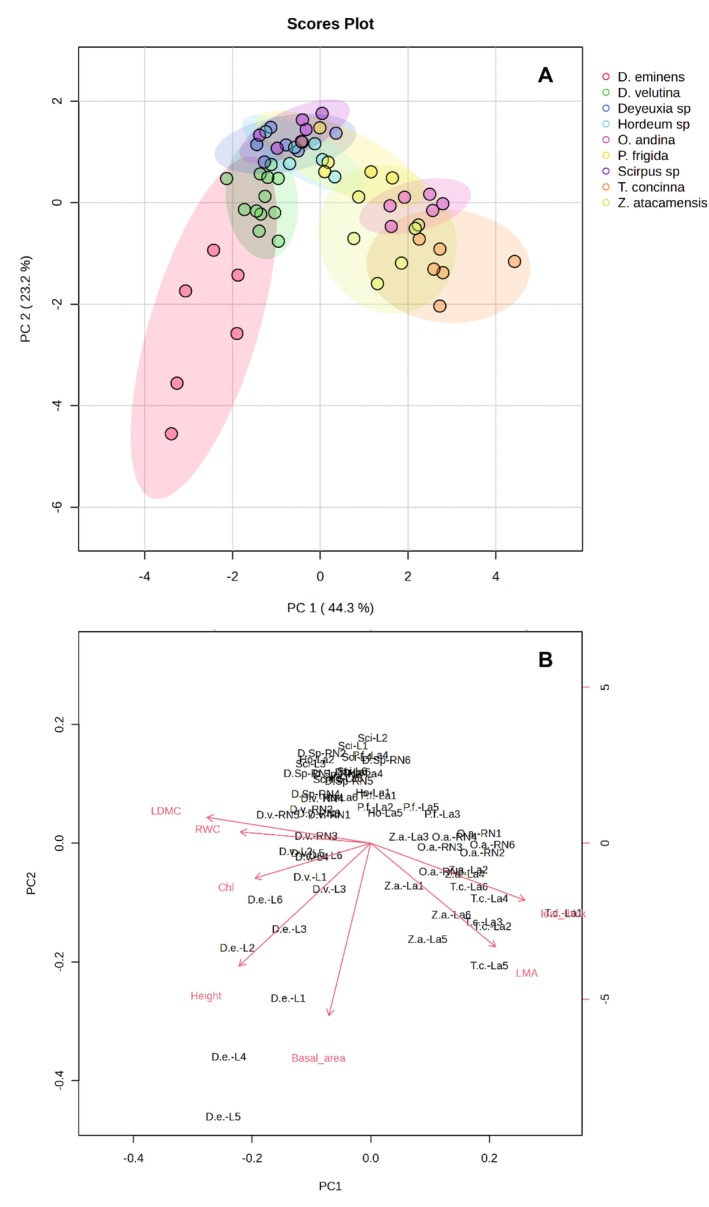
Analysis of principal components based on the morpho‐physiological traits of the evaluated species. The ordering of the individuals is shown in relation to the first two principal components (A) and the correlation of the variables with the ordering axes (B).

The PCA (Figure 2A) indicated that the first two axes accounted for 66.6% of the data variation, where PC1 explained 42.4% of the variation, while PC2 explained 24.2%. It is observed that the main differentiation between species occurred along PC1, where towards negative values of PC1 we have the grass species *D. eminens* and *D. velutina* , then towards less negative values *Deyeuxia sp*., *Scirpus sp*., and *P. frigida* .

Towards positive values were *Hordeum* sp., *Z. atacamensis*, and *O. andina* , with *T. concinna* showing the most positive values. Along PC2, the main differentiation was observed between *Scirpus* sp., which is located towards the most negative values, and *D. eminens* , which is located towards the most positive values. According to the PCA biplot (Figure 2B), it is observed that the morpho‐physiological traits responsible for the differentiation were LDMC, % RWC, and leaf thickness along PC1, where species such as *Z. atacamensis*, *T. concinna* , and *O. andina* have leaves with a higher LDMC than *Hordeum* and *Deyeuxia* species. Along PC2, the variation was associated with the SLA and the basal area of the plant, where *Scirpus* sp. had smaller plants with low SLA, while *D. eminens* presented larger individuals and high SLA values, although with greater individual variation. *T. concinna* and *Z. atacamensis* were also located at high SLA values. It was also observed that the same species but measured in different places, such as *D. velutina* (measured in the Leoncito and Río Negro HAWAs), did not show differences in its morpho‐physiological trait space.

**FIGURE 3:**
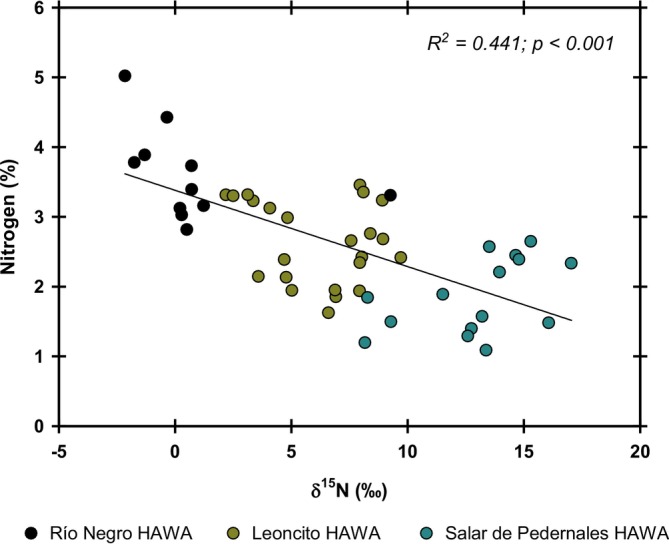
Significant correlation between δ^15^N and the leaf nitrogen percentage. Dots represent the corresponding values of nine different species (*n* = 5–6) distributed among the three HAWAs studied. The linear adjustment was the best fit showing the corresponding determination coefficient and *p‐*values.

Species with a leaf nitrogen percentage greater than 3% (*Deyeuxia* sp., *Scirpus* sp. and *O. andina* ) were only found in the HAWAs located at the head of the rivers (Leoncito and Río Negro). There was a linear and inversely proportional relationship between δ^15^N and the percentage of foliar nitrogen of the plants in these HAWAs (Figure 3). The highest δ^15^N and the lowest percentage of nitrogen were found in the vegetation that grows on the margins of the Salar de Pedernales lagoon.

**FIGURE 4:**
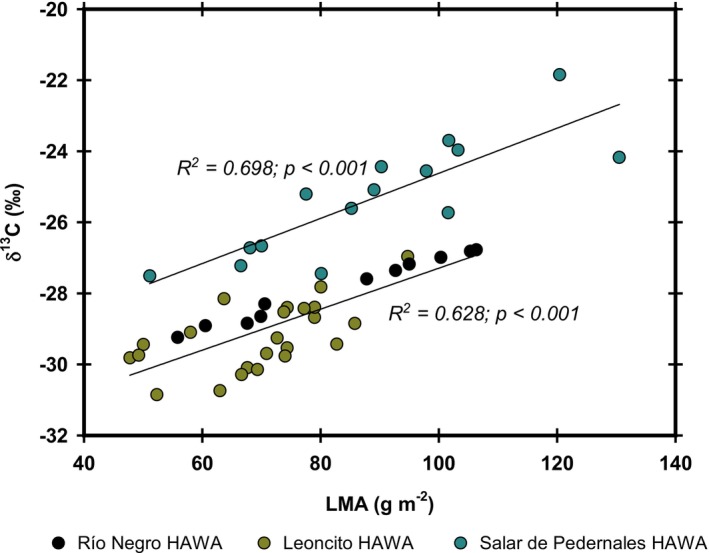
The relationship between δ^13^C and leaf mass area (LMA). Dots represent the corresponding values of nine different species (*n* = 5–6) distributed among the three HAWAs studied. The linear adjustment was the best fit showing the corresponding determination coefficient and *p‐*values. The upper regression line represents the relation between these variables in the three species living in the Salar de Pedernales HAWA, and the below regression line, represent the relation in the six remaining species from the Leoncito and Río Negro HAWAs.

Other variables positively correlated were the δ^13^C and the LMA (*R* ^*2*^ *= 0.577*; *p < 0.001*) of plant leaf tissue of all the HAWAs evaluated in the endorheic basin, with a δ^13^C increase of 0.084 C‰ g^−1^ m^2^. However, plants from the Salar de Pedernales HAWA usually presented higher δ^13^C values than those growing on the HAWAs located in the tributaries (Leoncito and Río Negro) (Figure 4). Subsequently, when the correlations between the δ^13^C and the LMA were analyzed independently for the Salar de Pedernales HAWA and the HAWAs located in the tributaries, both correlations improved (*R* ^*2*^ *= 0.698*; *p < 0.001* and *R* ^*2*^ *= 0.628*; *p < 0.001*, respectively), and remained highly significant. However, the δ^13^C increase rate was smaller, around 0.063 C‰ g^−1^ m^2^ for plants from the Salar de Pedernales, and 0.058 C‰ g^−1^ m^2^ for plants from the tributaries (Figure 4). This suggests that plants growing in a more saline environment discriminate less ^13^C at any LMA.

**FIGURE 5:**
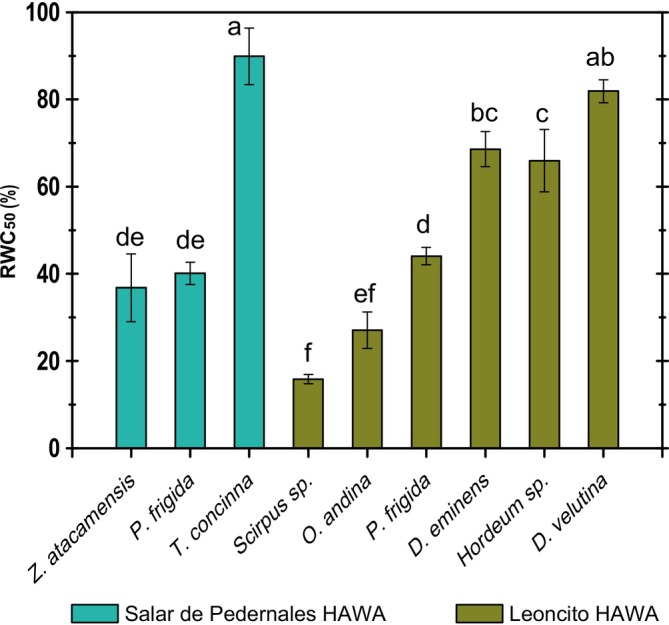
Dehydration tolerance of leaf tissue. Values represent the mean ± standard error (*n* = 3) of the relative water content at which 50% of photoinactivation occurs (RWC_50_) for the Salar de Pedernales and the Leoncito HAWAs plants. Significant differences among species are shown as different lower cases (*p* < 0.05).

Leaf dehydration tolerance, assessed as the RWC at which 50% of photoinactivation was attained, allowed for the identification of four species: *T. concinna* , *D. velutina* , *D. eminens* , and *Hordeum* sp., which presented a RWC_50_ significantly higher than the rest of the studied species (*F*_*1,18*_ *= 28.23; p < 0.001*). This group of species had 50% of photoinactivation by a small reduction in the RWC, with some of them being affected even above 70% of RWC (Figure 5). The rest of the species exhibited lower RWC_50_, with values below 50%, and the lowest value of RWC_50_ 15.8%, was recorded in *Scirpus* sp. at the Leoncito HAWA.

**FIGURE 6:**
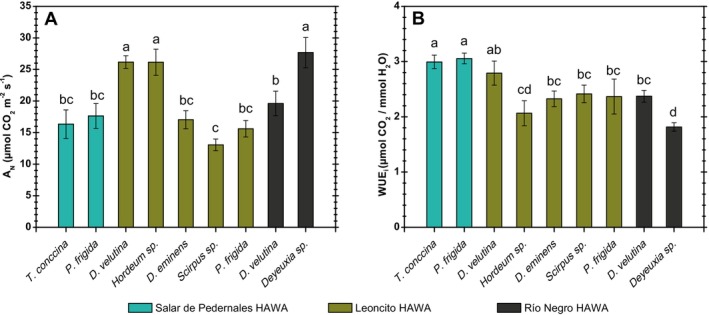
CO_2_ assimilation rate (AN) and instantaneous water use efficiency (WUEi) for the Salar de Pedernales, Leoncito and Río Negro HAWAs plants. Values represent the mean ± standard error (*n* = 6) of A_N_ i(A), and WUE_i_(B). Significant differences among species are shown as different lower cases (*p* < 0.05).

The species with the highest CO_2_ assimilation rate (A_N_) were found growing in the HAWAs present in the tributaries (Figure 6A) (from the Leoncito HAWA), with A_N_ higher than 25 μmol CO_2_ m^−2^ s^−1^; standing out significantly from the rest of the evaluated species (Figure 6A). On the other hand, the species measured in the Salar de Pedernales HAWA were those that had a significantly higher water use efficiency (WUEi) (Figure 6B). In addition, *Deyeuxia* sp. from Río Negro HAWA had the highest CO_2_ assimilation values, as well as the lowest WUEi values (Figure 6A,B).

**FIGURE 7:**
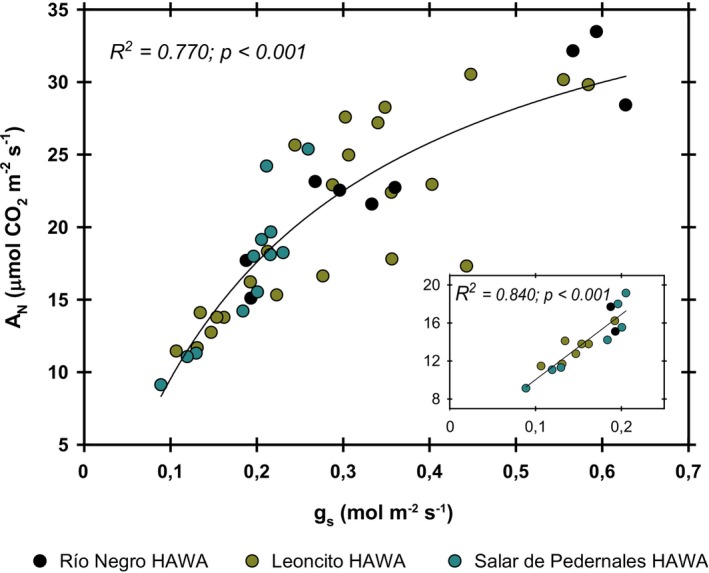
The relationship between net CO_2_ assimilation (A_N_) and stomatal conductance (g_s_). The dots represent the corresponding values of seven different species (*n* = 6) distributed among the three HAWAs studied. Regressions were selected as linear or simple hyperbolic adjustments based on the best fit, with the corresponding determination coefficient and *p‐*values.

There was a positive correlation between stomatal conductance (gs) and CO_2_ assimilation (A_N_) in the species of all the HAWAs analyzed (Figure 7). This relationship has a greater tendency towards linearity within a range of gs below 0.2 mol H_2_O m^−2^ s^−1^ (Figure 7), with an A_N_ increase of 0.068 μmol CO_2_ mol^−1^ H_2_O. The species from the Salar de Pedernales tended to be grouped towards the lowest range of stomatal conductance. This is why, even with an average net photosynthesis, they had the highest WUE_i_ (Figure 6B).

**FIGURE 8:**
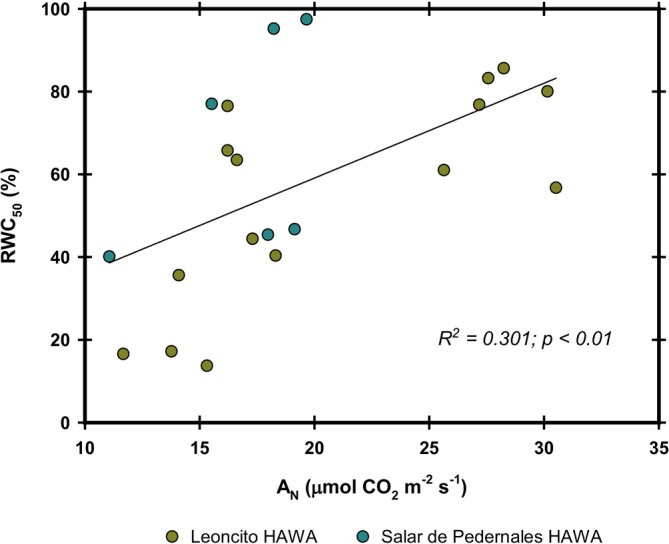
Correlation between relative water content (RWC50) and net CO_2_ assimilation (A_N_). The dots represent the corresponding values of seven different species (*n* = 6) distributed among the three HAWAs studied. Regressions were selected as linear or simple hyperbolic adjustments based on the best fit, with the corresponding determination coefficient and *p‐*values.

A positive correlation occurred between RWC_50_ and net CO_2_ assimilation among the species analyzed in the Salar de Pedernales and Leoncito HAWAs (Figure 8).

## 4 Discussion

The present study revealed that morpho‐physiological traits associated with photosynthetic gas exchange and leaf transpiration, including LDMC, %RWC, leaf thickness, and LMA, were the primary determinants of species‐level variation in the studied HAWAs. These findings also underscore the critical role of stomatal conductance in regulating CO_2_ assimilation. In contrast, chlorophyll content exhibited minimal influence on photosynthetic performance and did not explain the observed interspecific differences.

The negative relationship between foliar nitrogen percentage and δ^15^N suggests limited nitrogen availability from rock mineralization in the study area. None of the studied plant species is known to have a direct association with atmospheric nitrogen‐fixing microorganisms in their roots. Therefore, δ^15^N values closer to zero in plant species from Río Negro HAWA suggest greater microbial activity in the degradation of dead organic matter and/or the presence of diazotrophic organisms in the soil (Hobbie and Ouimette 2009).

It is known that nitrifying microbial communities, including species such as *Nitrosopumilus*, *Nitrosospira*, *Nitrosomonas*, *Kuenenia*, and *Nitrospira*, thrive in decreased salinity lagoons and reduce their activity when environmental salinity increases (Molina et al. 2018). The presence of these bacterial communities in the HAWAs located in the tributaries with low salinity (around 40.7 μS/cm) could explain why the plant communities in these areas exhibited nitrogen isotope compositions enriched in ^14^N isotopes, more similar to atmospheric isotopic compositions. In contrast, the higher salinity levels around 273 mS/cm in the Salar de Pedernales HAWA may result in plant nitrogen sources being richer in ^15^N isotopes. High salinity can inhibit microbial activity and slow the decomposition rate of soil organic matter due to osmotic stress (Chowdhury et al. 2011). Therefore, assuming that the release of assimilable nitrogen from mineralized soil nitrogen is a relatively homogeneous process throughout the endorheic basin, the higher percentage of foliar nitrogen with lower δ^15^N in plants from the Río Negro and Leoncito HAWAs could be attributed to a greater amount of assimilable nitrogen available in these sites. This availability is likely a result of enhanced nutrient recycling and atmospheric nitrogen fixation by microorganisms.

At the community level, the positive relationship between an increase in δ^13^C and LMA, as reported in the literature, is upheld (Lamont et al. 2002; Gerdol et al. 2018). An increase in LMA has been proposed to reduce CO_2_ diffusion through leaves, thereby lowering CO_2_ concentration in the chloroplast for photosynthesis. As a consequence of this CO_2_ limitation, Rubisco enzymes discriminate less against ^13^C (Takahashi and Miyajima 2008). Data analysis at the community level shows that for every g m^−2^ increase in LMA, δ^13^C increased by 0.084‰. However, plants growing in the Salar de Pedernales HAWA exhibit lower isotopic discrimination than those in the tributaries, suggesting that the higher salinity at this site influences physiological processes related to carbon assimilation, enhancing instantaneous water‐use efficiency (WUEi) and δ^13^C. This suggests that the actual variation rate of δ^13^C for these plants should range between 0.063‰ and 0.058‰ g^−1^ m^2^, lower values that account for the differences in salinity among the sites.

The HAWAs function as oases in the highland desert environment. These ecosystems have a close relationship with water availability, and most dominant plant species do not appear to have developed strategies to tolerate or resist dehydration. Approximately half of the species analyzed for dehydration tolerance lost leaf tissue functionality when the RWC decreased below 70%. Another group showed significant damage with RWC reductions to 40%–30%, while only one species, *Scirpus sp*., could be described as tolerant, maintaining functionality until RWC dropped to 20% before half of its photosynthetic tissue was visibly affected. The high dehydration‐tolerance of this species aligns with its morpho‐physiological traits such as small and thick leaves.

There was no clear pattern of dehydration tolerance associated with the studied sites. The most sensitive species, *T. concinna* from the Salar de Pedernales HAWA, exhibited 50% photoinactivation with about a 10% reduction in RWC. Similarly, in the Leoncito HAWA, three species ( *D. velutina* , *D. eminens* and *Hordeum* sp.) were also notably sensitive. Consistent with this lack of pattern, *P. frigida, a* species that coexists in both sites, had similar dehydration tolerance in both locations. As many mechanisms related to dehydration tolerance are also associated with salt tolerance, such as osmotic adjustment and the expression of LEA proteins of the dehydrin type (Hanin et al. 2011; Graether and Boddington 2014; Sun et al. 2021), it might be expected that plants growing in the Salar de Pedernales HAWA would have greater tolerance to dehydration than those growing at the Leoncito HAWA. However, there were no significant differences in the RWC_50_ of both communities, suggesting that the salinity tolerance mechanisms of the Salar de Pedernales species are independent of those related to dehydration tolerance present in other species.

Plants with the highest photosynthetic rates (*Deyeuxia* sp., *Hordeum* sp., and *D. velutina* ) were found growing in the HAWAs tributaries, while species with the highest water use efficiency grew in the Salar de Pedernales HAWA. Nevertheless, species from the Salar de Pedernales HAWA have photosynthetic rates similar to some of the species present in the tributaries. This indicates that salinity at the Salar de Pedernales HAWA is not acting as a limiting factor for carbon assimilation in those species. Consistent with findings by Flexas et al. (2013) in non‐stressed plants, a strong hyperbolic relationship between CO_2_ assimilation and g_s_ was observed (Figure 7). However, within the range of g_s_ between 0.1–0.2 mol H_2_O m^−2^ s^−1^, a strong linear relationship with photosynthesis was evident, strongly suggesting that in this range, g_s_ is the main factor determining CO_2_ assimilation in these species. These plants do not appear to be under severe water stress, as even in the Salar de Pedernales HAWA, g_s_ was greater than 0.1 mol H_2_O m^−2^ s^−1^. This is outside the critical range of 0.05–0.1 mol H_2_O m^−2^ s^−1^, where g_s_ is associated with oxidative stress and damage response (Flexas et al. 2006). This suggests that at the current stage, water is not a limiting factor for photosynthesis in the existing plants. Combined with the high RWC_50_ values in several of the species studied, this reaffirms the close relationship of these species with water availability in their environment. However, further reduction in water availability will likely cause severe impacts, especially in those species with high stomatal conductance, which could be more sensitive or vulnerable to dehydration. The inverse relationship observed between RWC_50_ and photosynthesis indicates that plants that have higher primary productivity are more susceptible to leaf dehydration damage in this species assembly. Therefore, as in other ecosystems, a trade‐off between productivity and stress tolerance is observed.

## 5 Conclusion

This study provides new insights into the physiological ecology of HAWA plant species. Morpho‐physiological traits, nitrogen cycling, and carbon isotope discrimination were shown to be influenced by environmental factors, including salinity and water availability. While the studied species have adapted to their challenging environment, their limited dehydration tolerance highlights their vulnerability to future hydrological changes. Understanding these physiological responses is crucial for developing effective conservation and management strategies to protect these valuable ecosystems.

## Conflicts of Interest

The authors declare no conflicts of interest.

## Supporting information


Data S1.

## Data Availability

Data collected from this experiment are available in the Supporting Information S1.
